# Kinematic Alignment Using Conventional Mechanical-Alignment Instruments with 3D-Printed Tibial Recut Guide Versus Manual Mechanical Alignment in Total Knee Arthroplasty: An IPTW-Adjusted Comparative Cohort Study

**DOI:** 10.3390/jcm15218558

**Published:** 2026-11-03

**Authors:** Bui Hong Thien Khanh, Pham Thi Cam Van, Nguyen Phuc Thinh

**Affiliations:** 1 Orthopaedic Department, University of Medicine and Pharmacy Ho Chi Minh City, Ho Chi Minh City 70000, Vietnam; khanh.bht@umc.edu.vn; 2 Department of Radiology, Diagnostic Medical Center Medic, Ho Chi Minh City 70000, Vietnam; bscamvanmedic@gmail.com; 3 Orthopaedic Department, University Medical Center Ho Chi Minh City, Ho Chi Minh City 70000, Vietnam

**Keywords:** total knee arthroplasty, kinematic alignment, mechanical alignment, inverse probability of treatment weighting, Knee Society Score

## Abstract

**Background/Objectives**: Unrestricted kinematic alignment (KA) may produce excessive tibial varus in some knees. We evaluated a modified KA technique using conventional mechanical instruments combined with a standardized 3D-printed 2° tibial recut guide that retained calipered femoral resurfacing while selectively restricting tibial varus, comparing it with historical manual mechanical alignment (MA) controls. **Methods**: This single-centre comparative cohort study included 120 knees from 104 patients (66 consecutive prospective KA knees and 54 historical MA knees). Inverse probability of treatment weighting targeting the average treatment effect on the treated adjusted for age, sex, body mass index, ASA class, and preoperative hip–knee–ankle angle. Treatment-effect inference used patient-level cluster-robust standard errors across 104 patient clusters. Outcomes included the 2011 Objective Knee Society Score (Objective KSS), patient-reported outcomes, and postoperative coronal alignment. **Results**: The recut guide was used in 37/66 KA knees (56.1%). KA was associated with a higher Objective KSS (ATT +15.47; 95% CI 9.07–21.87; *p* < 0.001), driven mainly by alignment (+11.93; 95% CI 5.93–17.94; *p* < 0.001) and stability (+2.16; 95% CI 0.54–3.79; *p* = 0.009), while the Objective KSS ROM component did not differ significantly. Patient-reported outcomes were not significantly different, including WOMAC (ATT −4.16; 95% CI −8.33 to 0.02; *p* = 0.051), OKS (ATT −1.89; 95% CI −3.88 to 0.10; *p* = 0.062), FJS (ATT +10.70; 95% CI −1.31 to 22.71; *p* = 0.081), and total KSS (ATT +5.17; 95% CI −2.36 to 12.70; *p* = 0.179). KA showed less residual varus HKA (−2.03° vs. −4.61°), fewer postoperative HKA outliers beyond ±6° (6.1% vs. 38.9%), higher FMA (91.20° vs. 89.26°), and a more horizontal joint line, whereas TMA did not differ significantly (*p* = 0.331). **Conclusions**: Modified KA with selective tibial varus restriction was associated with a higher postoperative Objective KSS and more favorable coronal alignment than historical manual MA, while patient-reported outcomes were largely comparable. These findings support the technical feasibility of the approach but do not establish causal or clinical superiority over contemporary MA.

## 1. Introduction

Mechanical alignment (MA) remains a widely used method in total knee arthroplasty (TKA), aiming to position the femoral and tibial components relative to their mechanical axes and restore a near-neutral limb axis. However, native coronal knee anatomy varies considerably, and constitutional varus is present in a substantial proportion of asymptomatic non-arthritic individuals [1]. Moreover, when MA is performed with conventional manual instruments, control of the intended neutral mechanical axis is less consistent than with computer navigation or robotic assistance, which both reduce the frequency of coronal alignment outliers [2]. In knees with severe varus deformity, achieving neutral mechanical alignment can be particularly challenging because asymmetric bone loss and medial soft-tissue contracture may require greater tibial resection and more extensive soft-tissue balancing [3].

Kinematic alignment (KA) was developed to restore the estimated pre-arthritic joint surfaces through individualized resurfacing [4]. A concern with unrestricted KA, however, is that tibial resurfacing may reproduce excessive varus beyond the range of constitutional varus typically observed in non-arthritic knees, particularly in patients with marked varus deformity. Several methods have therefore been proposed to constrain personalized alignment. Blakeney and Vendittoli introduced restricted KA, limiting both femoral and tibial component orientations to within approximately ±5° of their respective mechanical axes and the final HKA to within ±3° of neutral [5,6,7]. Matsumoto described a tibia-restricted modified KA method using a systematic 3° varus tibial cut while maintaining unrestricted KA principles on the femoral side [8]. Winnock described inverse restricted KA, a tibia-first method in which the tibial joint line is restored first and subsequent femoral resections are adjusted to balance the knee [9,10]. These approaches differ in the degree and manner of alignment restriction and have been implemented using computer-assisted technologies, including navigation or robotic systems, to control component orientation.

In the present study, KA was performed predominantly with conventional mechanical-alignment instruments. Femoral resections followed Howell’s calipered resurfacing principles [11,12,13], whereas excessive tibial varus was selectively controlled using a standardized 3D-printed recut guide providing an additional 2° correction per application, used no more than twice, with the practical aim of avoiding tibial varus beyond approximately 5°. This approach combines the individualized principles of KA with conventional instruments and may be more accessible in settings where navigation or robotic systems are not routinely available.

We therefore compared this modified KA technique with conventional manual MA using inverse probability of treatment weighting. The primary outcome was the postoperative 2011 Objective Knee Society Score, while secondary outcomes included other clinical, patient-reported, and radiographic measures. We hypothesized that the modified KA technique would result in a higher postoperative Objective KSS, better patient-reported outcomes, and more favorable radiographic alignment than conventional manual MA.

## 2. Materials and Methods

### 2.1. Study Design and Participants

This was a single-centre comparative cohort study comprising a prospectively enrolled consecutive KA cohort and a historical MA control cohort. The ethics-approved study protocol specified the use of the historical MA cohort as a control for postoperative assessment. The KA cohort consisted of consecutive knees undergoing primary TKA with the modified KA from July 2022 to December 2025. The historical MA cohort comprised knees from patients who underwent surgery between January 2020 and July 2022; their postoperative outcomes were assessed as specified in the approved research protocol. Thus, the overall study data covered the period from July 2022 to December 2025 for research assessment, while the historical MA surgeries had been performed before ethics approval. All procedures were performed by the same surgical team using the same implant system, a cemented Medacta total knee prosthesis with an ultra-congruent mobile-bearing polyethylene insert.

Patients were eligible if they had primary Kellgren–Lawrence grade 4 knee osteoarthritis on standing radiographs, persistent symptoms despite non-operative treatment, and an indication for primary TKA. Additional eligibility criteria included the absence of substantial distal femoral bone loss, collateral-ligament injury, quadriceps- or patellar-tendon injury, inflammatory arthropathy, or periarticular bone tumour. Patients were excluded if progressive neurological or neuromuscular disease interfered with reliable postoperative functional assessment or if the required postoperative clinical and full-length radiographic evaluation could not be completed.

**Figure 1:**
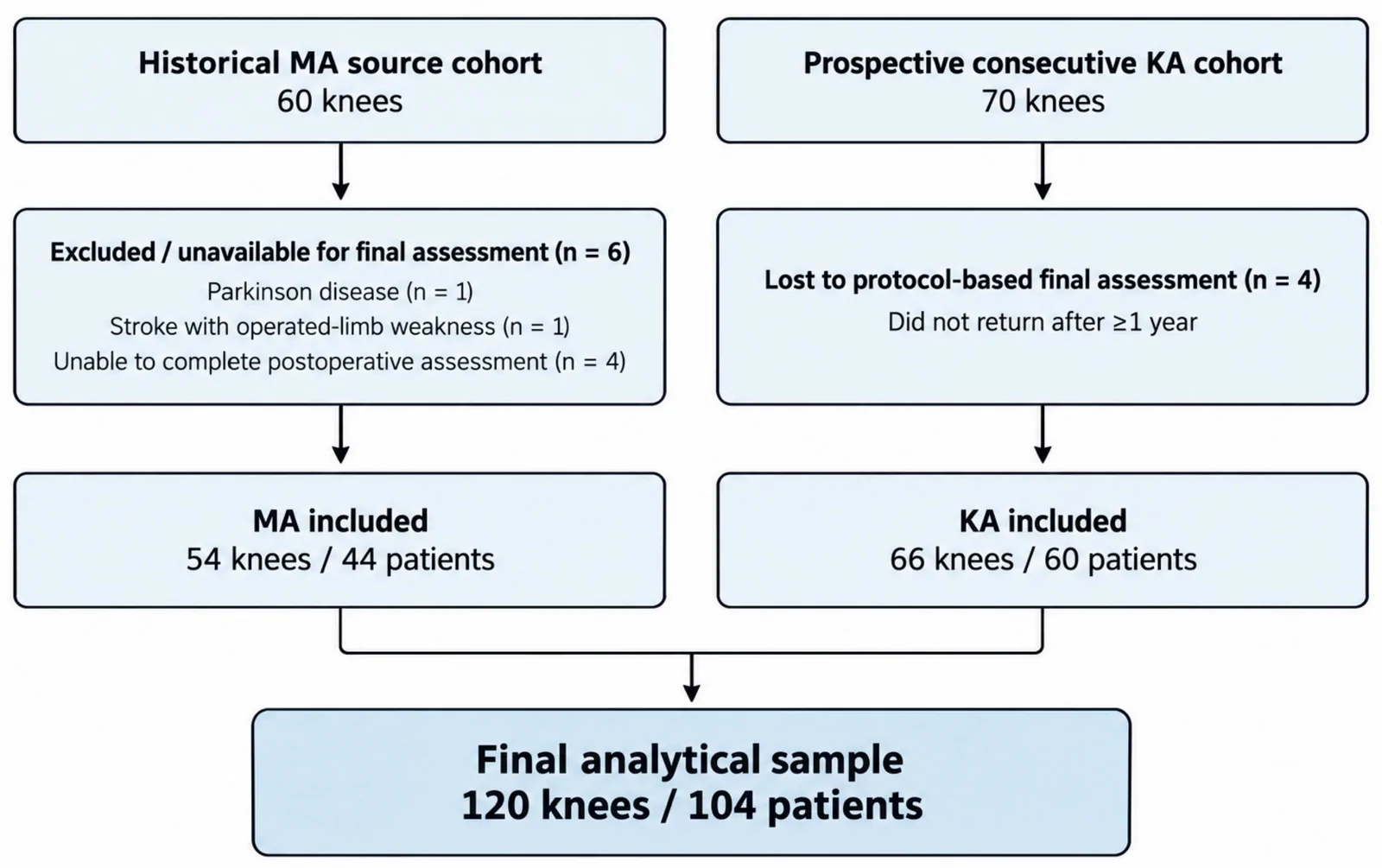
Study flow diagram.

The historical MA source cohort included 60 knees with preoperative and postoperative EOS imaging. One knee was excluded because of progressive Parkinson disease, one because of stroke causing weakness of the operated limb, and four because postoperative assessment could not be completed, leaving 54 knees in 44 patients. Seventy consecutive KA knees were prospectively enrolled; four did not return for protocol-based final assessment after at least 1 year and were considered lost to follow-up, leaving 66 knees in 60 patients. The final analytical sample therefore comprised 120 knees in 104 patients. Sixteen patients underwent bilateral TKA, including 10 patients in the MA group and 6 patients in the KA group; the remaining patients underwent unilateral TKA (Figure 1).

### 2.2. Sample Size

The sample-size calculation was based on the postoperative 2011 Objective Knee Society Score (Objective KSS) [8]. Because the historical MA cohort had been established before the recruitment of the KA cohort, the MA sample was fixed at 54 knees. Based on the postoperative Objective KSS reported by Matsumoto et al. (95.0 ± 4.2 for KA and 92.8 ± 4.3 for MA), a two-sided α of 0.05 and 80% power were specified. A minimum of 65 KA knees was required; a total of 66 KA knees were ultimately included.

### 2.3. Surgical Procedures

#### 2.3.1. Mechanical Alignment

Both groups underwent the same surgical exposure. In the MA group, the distal femoral resection was performed using an intramedullary guide with a fixed 6° valgus setting and a 9-mm resection target. The proximal tibial resection was performed with an extramedullary guide aiming for a cut perpendicular to the tibial mechanical axis. The remaining femoral cuts were performed using 3° of external rotation. Flexion and extension gaps were balanced using selective soft-tissue release, additional tibial or distal femoral resection when required, and adjustment of femoral component size.

#### 2.3.2. Kinematic Alignment with Selective Tibial Varus Restriction

The KA procedure used the same surgical exposure and implant system as the MA procedure but followed a modified kinematic alignment workflow. Femoral resections were determined by calipered resurfacing principles based on estimated pre-arthritic anatomy, whereas tibial restriction was introduced only to avoid excessive varus beyond the predefined limit.

**Figure 2:**
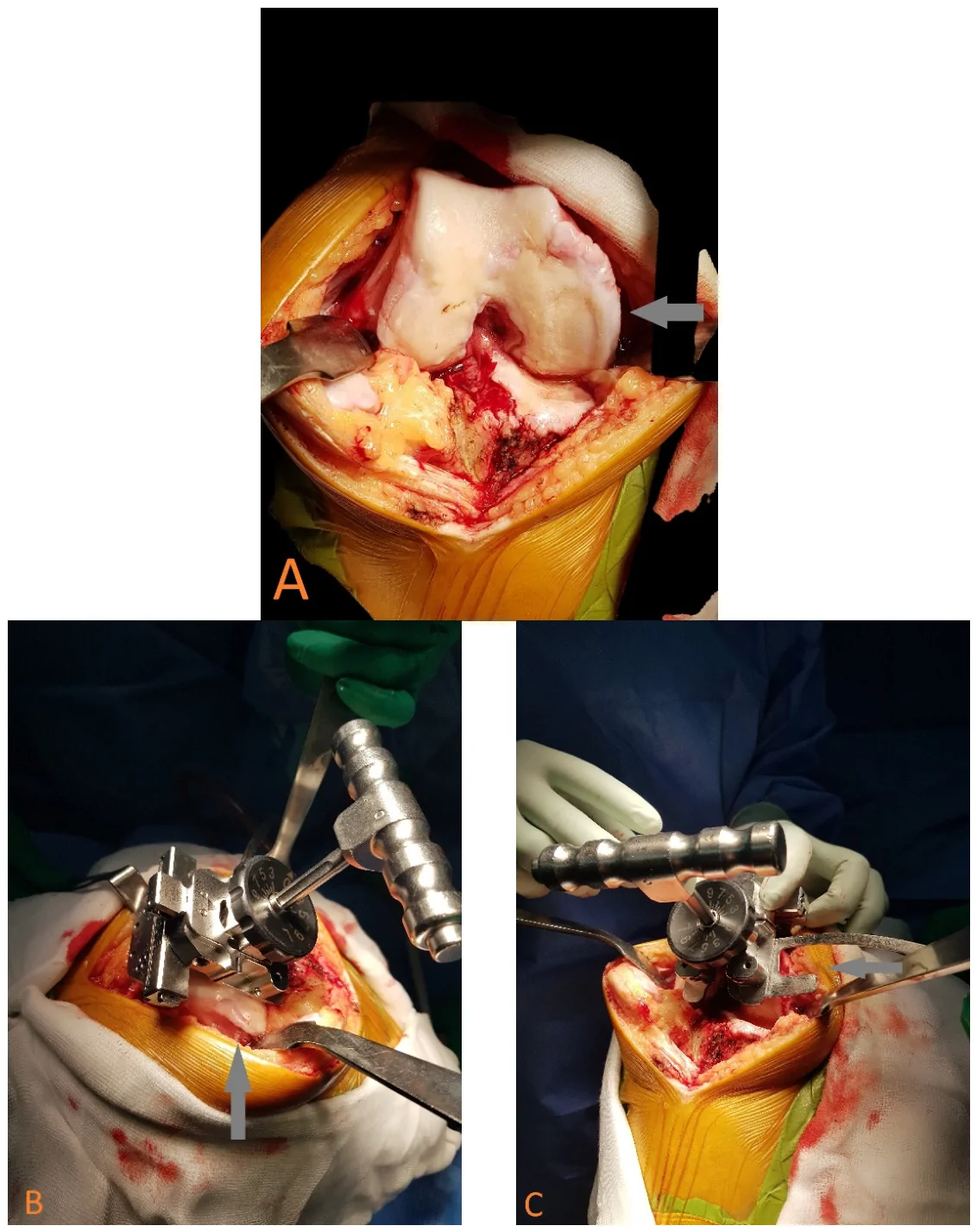
Compensation for distal femoral cartilage loss and intramedullary distal femoral resection. (**A**) Typical varus osteoarthritic right knee. (**B**) Intact lateral cartilage (Gray arrow). (**C**) Worn medial condyle (Gray arrow).

Articular cartilage status was assessed intraoperatively before bone resection. Residual cartilage and areas of full-thickness cartilage loss were identified by making a small incision or mark through the articular surface with a scalpel or electrocautery until the subchondral bone was identified. For the distal femur, the cutting guide was referenced to preserved cartilage. After the removal of the peripheral osteophytes, in the typical varus osteoarthritic knee with preserved lateral condylar cartilage and full-thickness medial cartilage loss (Figure 2A), the guide was placed against the intact lateral cartilage (Figure 2B), while a 2-mm spacer angle wing was applied to the worn medial condyle to compensate for the estimated loss of cartilage thickness (Figure 2C). The intended distal femoral resection thickness was 9 mm, corresponding to the distal femoral component thickness. The intramedullary valgus orientation was individualized rather than fixed at the conventional 6° MA setting; the intramedullary entry opening could be widened mediolaterally to permit the controlled adjustment of the cutting-guide orientation.

All patients underwent primary total knee arthroplasty using the GMK Primary system (Medacta International, Switzerland) with a mobile-bearing ultra-congruent (UC) polyethylene insert. The posterior cruciate ligament was sacrificed in all procedures, and patellar resurfacing was not performed. The same implant system, polyethylene insert design, and perioperative care protocol were used in both the MA and KA cohorts; the primary difference between the cohorts was the bone-resection and alignment technique used.

**Figure 3:**
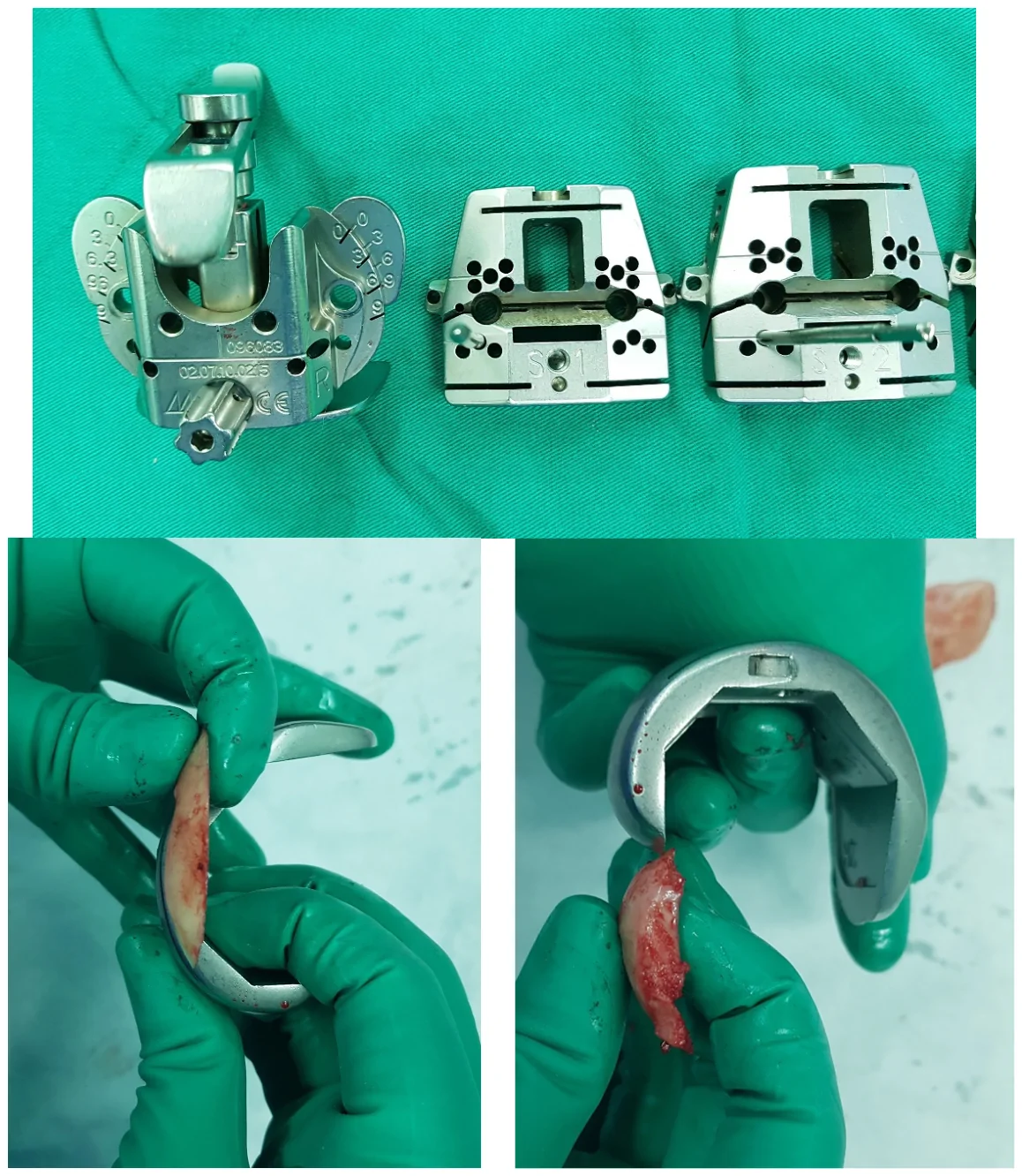
Posterior-referencing femoral preparation and corresponding prosthetic component thickness.

Femoral sizing and the anterior, posterior, and chamfer resections were performed using a posterior-referencing guide at 0° external rotation (Figure 3). Posterior cartilage loss, when present, was compensated for in the same manner before posterior femoral resection. Distal and posterior femoral resection thicknesses were verified with a caliper, taking into account the estimated cartilage loss and saw-blade thickness, to reproduce the corresponding prosthetic component thickness (Figure 3).

**Figure 4:**
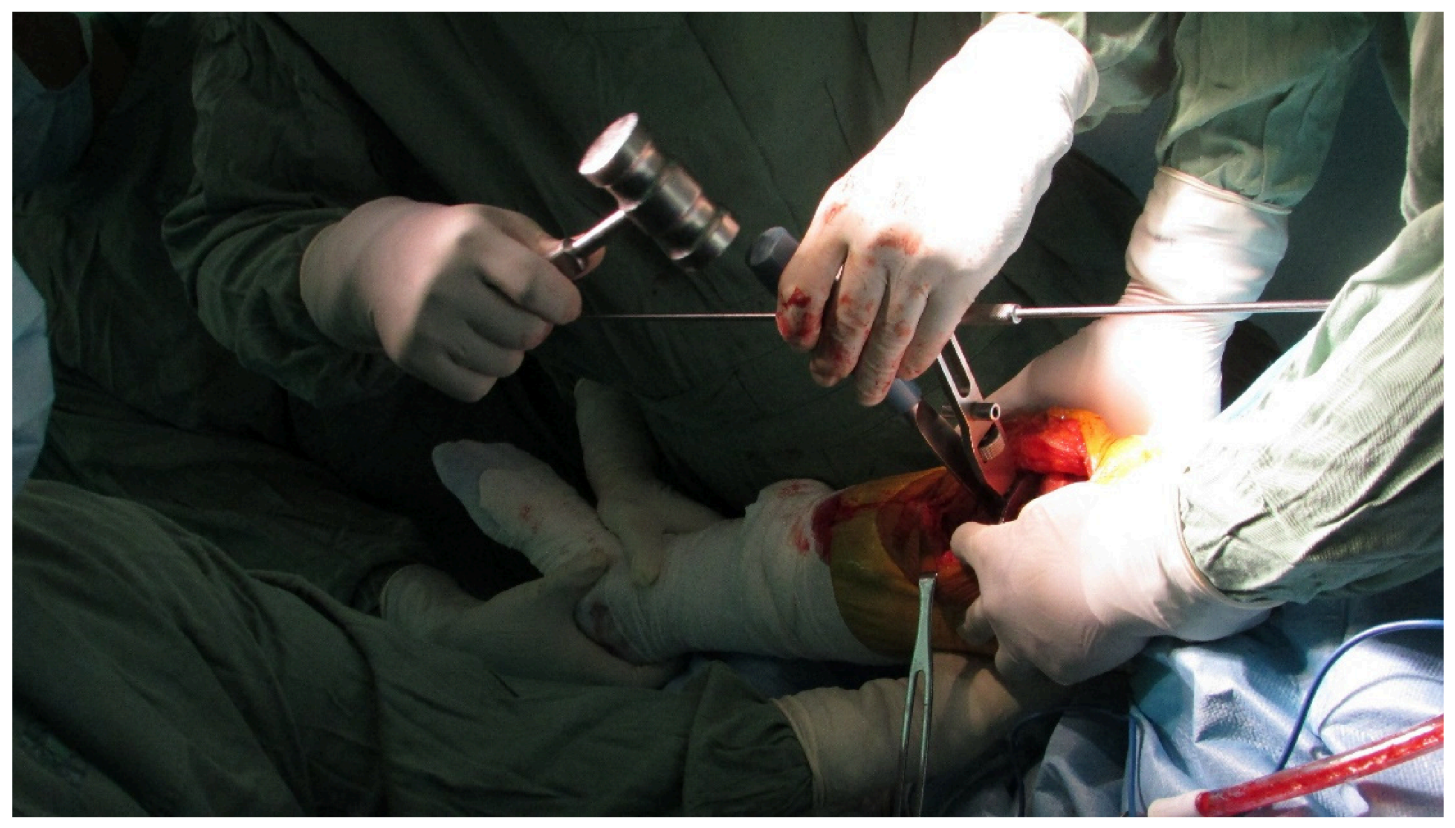
Tibial resection plane marked with the lower limb straightened under tension.

After the completion of the distal femoral resection, the limb was brought into extension under straightened tension and a spacer block was used to determine the initial tibial resection plane (Figure 4). In varus knees, the minimum tibial resection was referenced to the less affected lateral plateau. Tibial resection depth, posterior slope, and coronal orientation were subsequently adjusted according to flexion–extension gap balance. The initial posterior tibial slope was cut parallel to the sagittal inclination of the medial tibial plateau; if the flexion gap remained tight, the posterior tibial slope was increased by the smallest amount necessary to achieve soft-tissue balance.

**Figure 5:**
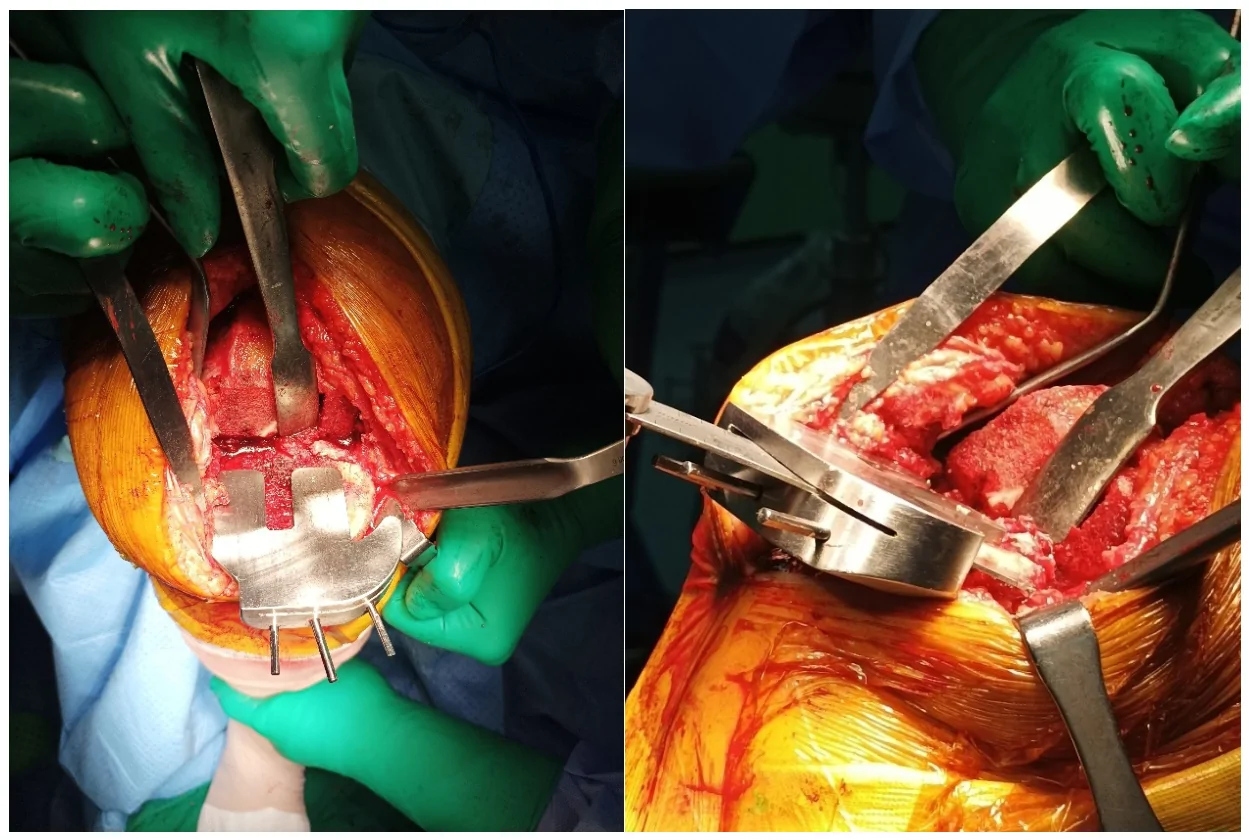

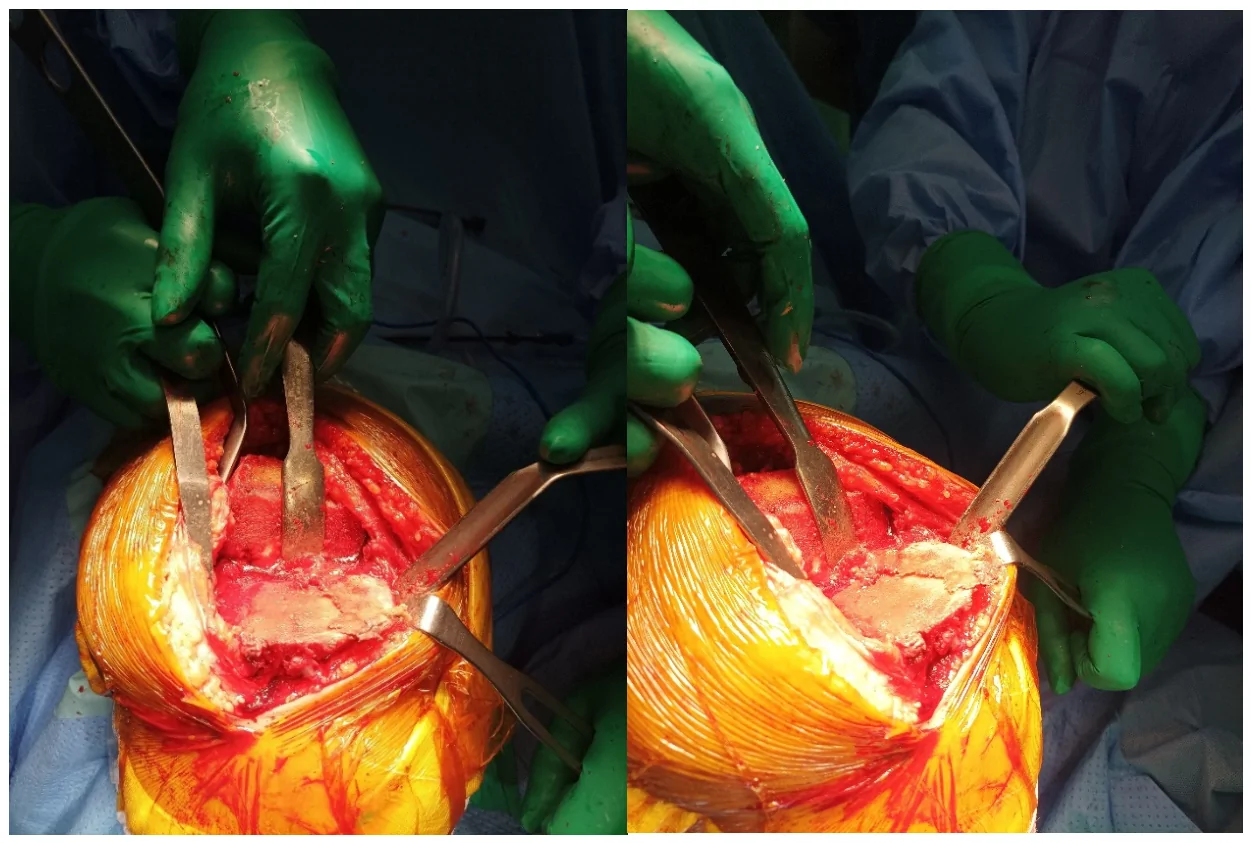
Three-dimensional printed 2° varus tibial recut guide used for controlled tibial recutting.

**Figure 6:**
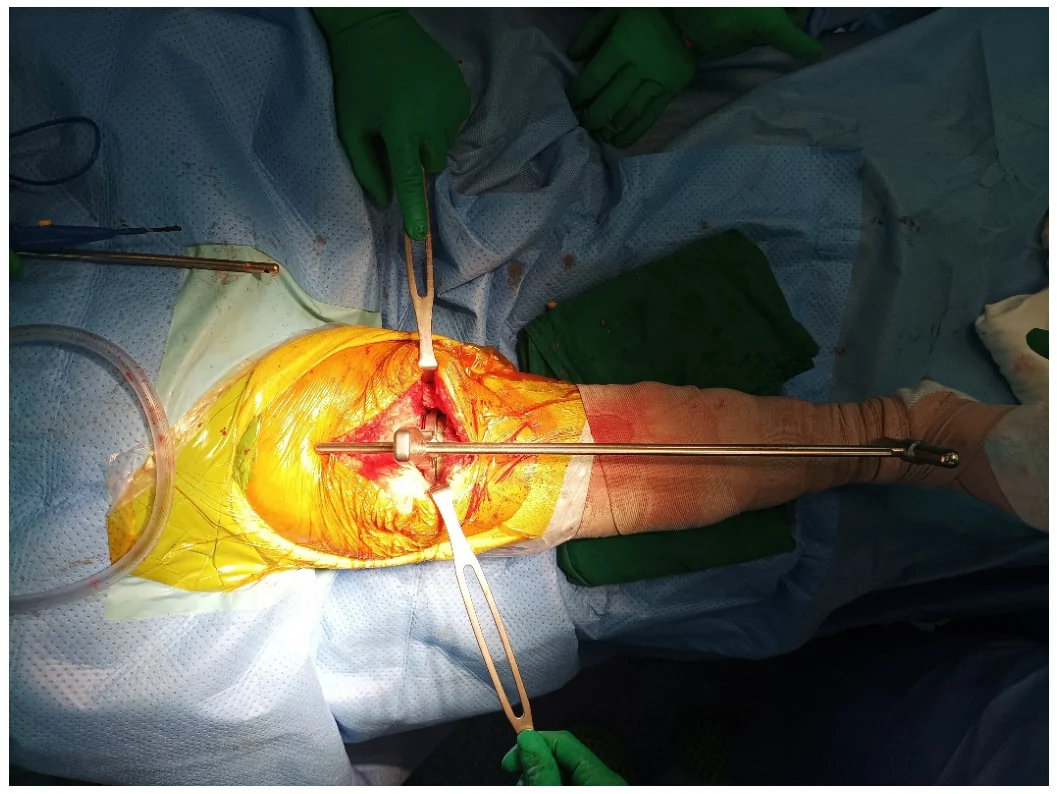
Intraoperative limb alignment assessment using a spacer block and alignment rod.

When the medial flexion and/or extension gap remained tight, the medial and lateral gaps were compared by assessing the fit of spacer blocks of appropriate thickness in both compartments, with the knee in 90° flexion and an assistant supporting the thigh for the assessment of the flexion gap, and with the knee in 0° extension and an assistant applying axial traction through the ankle for the assessment of the extension gap. If the medial compartment remained tighter than the lateral compartment, additional tibial varus was introduced in a controlled manner using a standardized 3D-printed 2° tibial recut guide (Figure 5). Each application increased the tibial varus angle by 2° relative to the preceding resection plane. The guide was used no more than twice, thereby limiting the intended tibial varus to approximately 5°. Limb alignment was manually checked intraoperatively using a spacer block with an alignment rod (Figure 6). Thus, femoral alignment remained based on calipered kinematic resurfacing, whereas tibial varus was selectively adjusted and restricted using the 3D-printed recut guide.

### 2.4. Perioperative Management

Both groups followed the same enhanced-recovery perioperative pathway. A thigh tourniquet was routinely used. Intravenous tranexamic acid and cefazolin prophylaxis were administered before tourniquet inflation, and topical tranexamic acid was used before wound closure. Periarticular multimodal analgesic infiltration was performed intraoperatively. Drains were removed within 24 h. Knee-motion exercises began on the day of surgery, and assisted ambulation with a walking frame began on the first postoperative day.

### 2.5. Outcome Assessment

Postoperative outcomes were assessed 12–24 months after surgery. Clinical outcomes were assessed by an independent evaluator who was not involved in the surgical procedure. Clinical outcomes included the 2011 Objective KSS and knee range of motion (ROM). Patient-reported outcomes included the Oxford Knee Score (OKS), the Western Ontario and McMaster Universities Osteoarthritis Index (WOMAC), the Forgotten Joint Score (FJS), and the patient-reported domains of the 2011 Knee Society Score, including symptom, satisfaction, expectation, function, and total scores.

The 2011 Objective Knee Society Score (Objective KSS) was calculated as the sum of the surgeon-assessed Objective KSS component and the KSS symptom score. The surgeon-assessed Objective KSS component comprised three domains: alignment, stability, and range of motion (ROM). The alignment component could yield negative points according to the degree of residual malalignment. The ROM component was scored according to the measured range of motion, with deductions applied for residual flexion contracture or hyperextension, where applicable.

**Figure 7:**
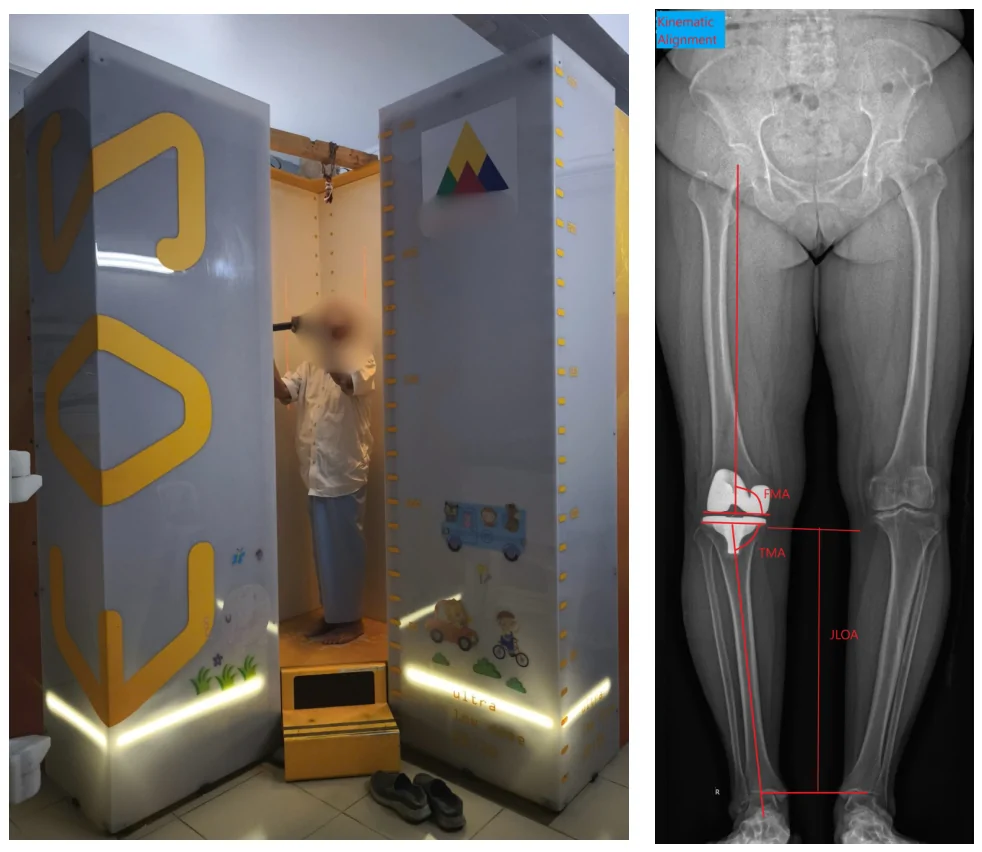
Radiographic EOS measurements of femoral mechanical angle (FMA), tibial mechanical angle (TMA), and joint-line obliquity angle (JLOA).

Standing full-length EOS biplanar (extra-low-dose X-ray imaging) radiographs were used for coronal alignment assessment, with three-dimensional (3D) reconstruction and measurements performed from the biplanar images (Figure 7). All radiographic measurements were performed once by the same experienced radiologist from another center, who had extensive experience in musculoskeletal imaging. The radiographic assessor was not involved in the surgical procedures. HKA was defined from the mechanical axes of the femur and tibia and was coded negative for varus and positive for valgus. Femoral mechanical angle (FMA) and tibial mechanical angle (TMA) were measured using the study’s 90° component-reference convention; higher FMA values indicated a more valgus distal femoral component orientation, whereas lower TMA values indicated greater tibial varus. The joint-line obliquity angle (JLOA) was measured relative to the horizontal reference. Postoperative HKA outliers were predefined as HKA deviations beyond ±6° [14].

### 2.6. Propensity Score Weighting and Statistical Analysis

Statistical analyses were performed using SPSS version 22.0 and Stata version 17.0. Propensity scores were estimated using logistic regression including age, sex, body mass index (BMI), the American Society of Anesthesiologists (ASA) physical status classification, and preoperative HKA. Inverse probability of treatment weighting (IPTW) was applied to improve balance in measured baseline characteristics.

The estimand was the average treatment effect on the treated (ATT), with KA defined as the treated group. KA knees received a weight of 1, whereas MA knees received PS/(1−PS), where PS was the estimated probability of receiving KA. Covariate balance was assessed using standardized mean differences (SMDs), with an absolute SMD < 0.10 considered acceptable. Weighted regression models were used to estimate adjusted KA–MA differences with 95% confidence intervals (CIs) and two-sided *p* values. Categorical radiographic outcomes were expressed as adjusted risk differences where appropriate. Because some patients contributed bilateral knees, robust standard errors were clustered at the patient level using the corrected composite patient identifier (group × original ID), yielding 104 patient clusters across 120 knee-level observations. Statistical significance was defined as *p* < 0.05. The knee was the unit of analysis for descriptive and weighting procedures, while treatment-effect inference accounted for within-patient clustering.

### 2.7. Ethics

The study was approved by the Institutional Biomedical Research Ethics Committee of the University Medical Center Ho Chi Minh City (approval No. 82/GCN-HĐĐĐ). The ethics certificate was formally signed on 29 September 2022 and specified the approved research implementation period as July 2022 to December 2025. Written informed consent was obtained from participants prospectively enrolled in the KA cohort.

## 3. Results

### 3.1. Study Population and Baseline Characteristics

A total of 120 knees from 104 patients were included in the final analysis: 54 knees in 44 patients in the historical MA group and 66 knees in 60 patients in the prospective consecutive KA group. Final outcome assessment was unavailable for 6 of 60 MA knees (10.0%) and 4 of 70 KA knees (5.7%). Sixteen patients underwent bilateral TKA, including 10 patients in the MA group and 6 patients in the KA group; the remaining patients underwent unilateral TKA.

Baseline characteristics were broadly similar between groups on conventional hypothesis testing. Mean age was 65.3 ± 8.2 years in the MA group and 67.2 ± 7.6 years in the KA group; mean BMI was 25.7 ± 2.8 and 25.6 ± 2.6 kg/m^2^, respectively. Both cohorts had marked preoperative varus alignment, with mean HKA values of −13.7 ± 8.9° in MA and −13.9 ± 6.5° in KA (Table 1).

The mean postoperative follow-up duration was 18.06 ± 4.77 months in the MA group and 18.50 ± 4.05 months in the KA group. The median follow-up duration was 18 months in both groups, with a range of 12–24 months. No statistically significant difference in follow-up duration was observed between groups (Welch’s *t*-test, *p* = 0.588).

Among the 66 KA knees, the 3D-printed 2° tibial recut guide was used in 37 knees (56.1%). Thirty-six knees (54.5%) required one recut, one knee (1.5%) required two recuts, and twenty-nine knees (43.9%) did not require the guide.

### 3.2. Covariate Balance After IPTW

**Figure 8:**
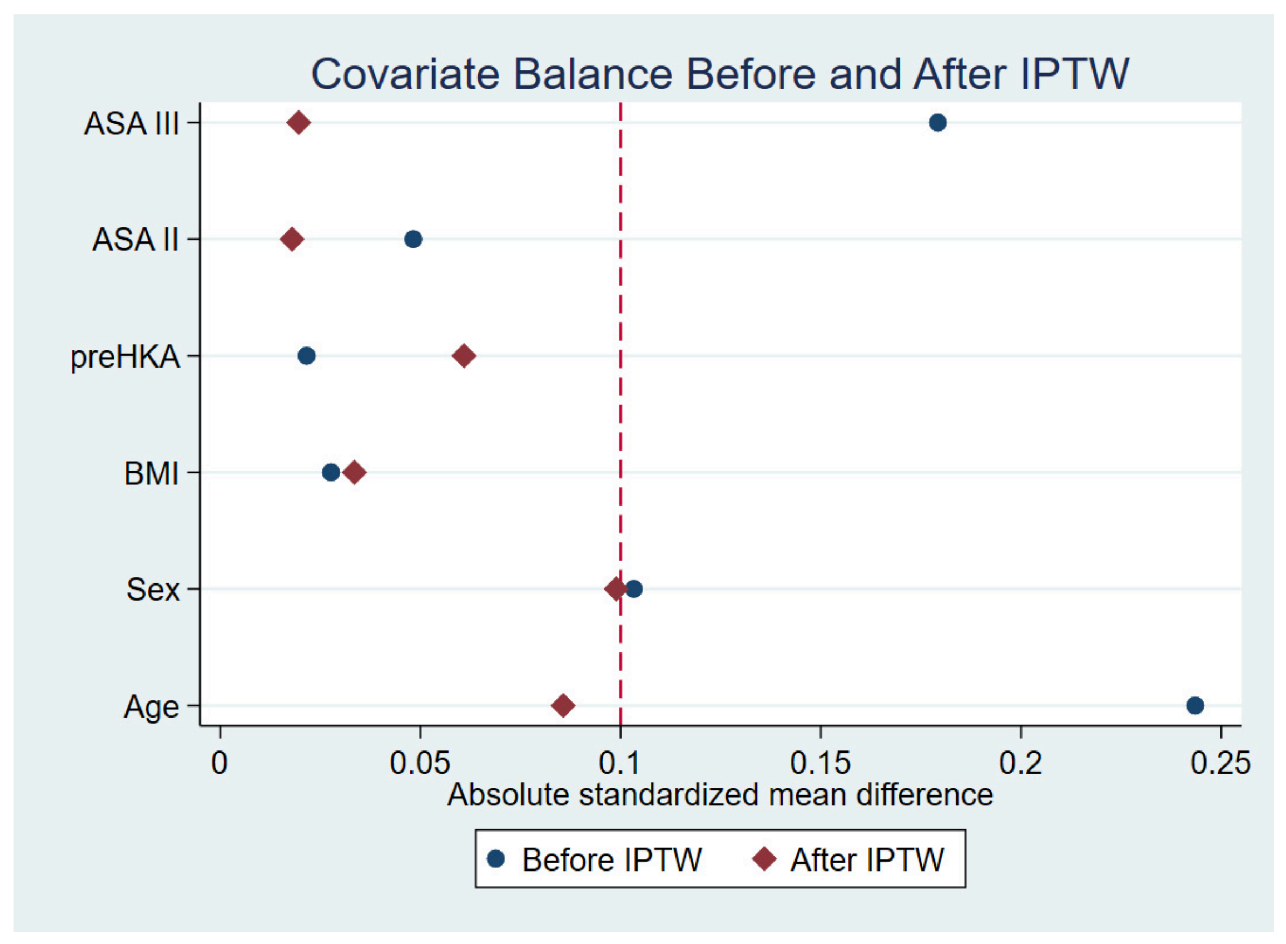
Covariate balance (Love plot). Absolute standardized mean differences before and after IPTW–ATT; the dashed line indicates the conventional 0.10 threshold.

**Figure 9:**
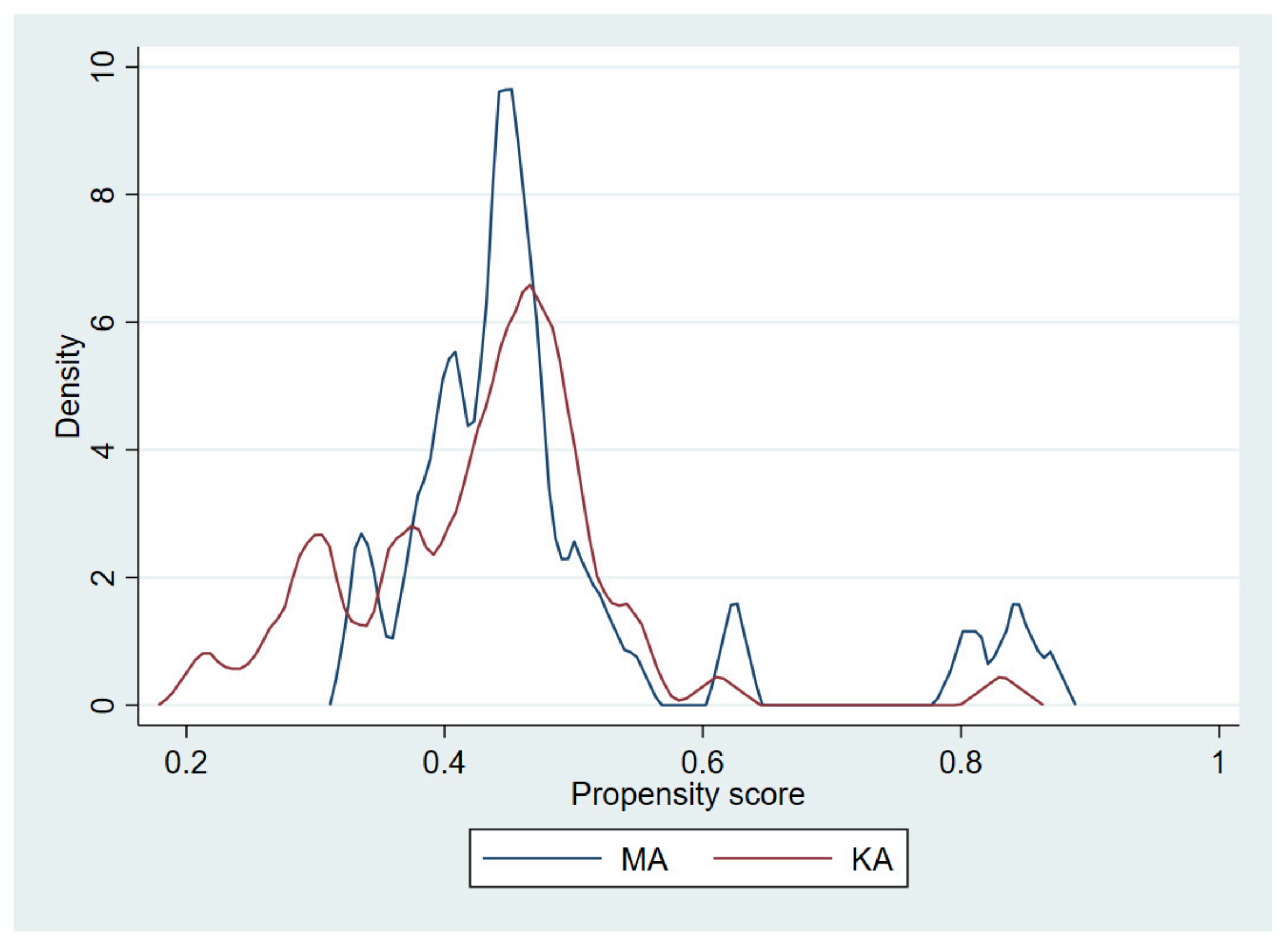
Propensity score overlap.

The propensity-score model included age, sex, BMI, ASA classification, and preoperative HKA. Before weighting, the largest absolute standardized mean differences (SMDs) were observed for age (0.244), ASA class III (0.179), and male sex (0.103). After IPTW targeting of the average treatment effect on the treated (ATT), all six assessed covariate indicators had absolute SMDs below 0.10 (range, 0.018–0.099), indicating adequate balance in the measured baseline covariate means and proportions (Table 2) (Figure 8). The weighted variance ratio for preoperative HKA remained 0.593, suggesting residual differences in covariate dispersion. Visual inspection of the propensity-score distributions showed substantial overlap in the central region, with differences in the tails (Figure 9).

### 3.3. Objective KSS and Range of Motion

The postoperative Objective KSS 2011 was 90.02 ± 12.74 in the KA group and 75.76 ± 19.14 in the MA group. After IPTW adjustment with patient-level clustering, KA was associated with a 15.47-point higher Objective KSS (95% CI, 9.07 to 21.87; *p* < 0.001). The surgeon-assessed Objective KSS component was also higher in KA (ATT, +14.46 points; 95% CI, 8.34 to 20.58; *p* < 0.001), with the largest component-level difference in alignment (ATT, +11.93 points; 95% CI, 5.93 to 17.94; *p* < 0.001), followed by stability (ATT, +2.16 points; 95% CI, 0.54 to 3.79; *p* = 0.009). The Objective KSS ROM component did not differ significantly between groups (ATT, +0.37 points; 95% CI, −0.79 to 1.53; *p* = 0.534). Measured postoperative ROM was likewise similar (112.73 ± 11.13° in KA vs. 112.04 ± 16.56° in MA; ATT, +0.98°; 95% CI, −4.99 to 6.96; *p* = 0.747) (Table 3).

### 3.4. Patient-Reported Outcomes

**Figure 10:**
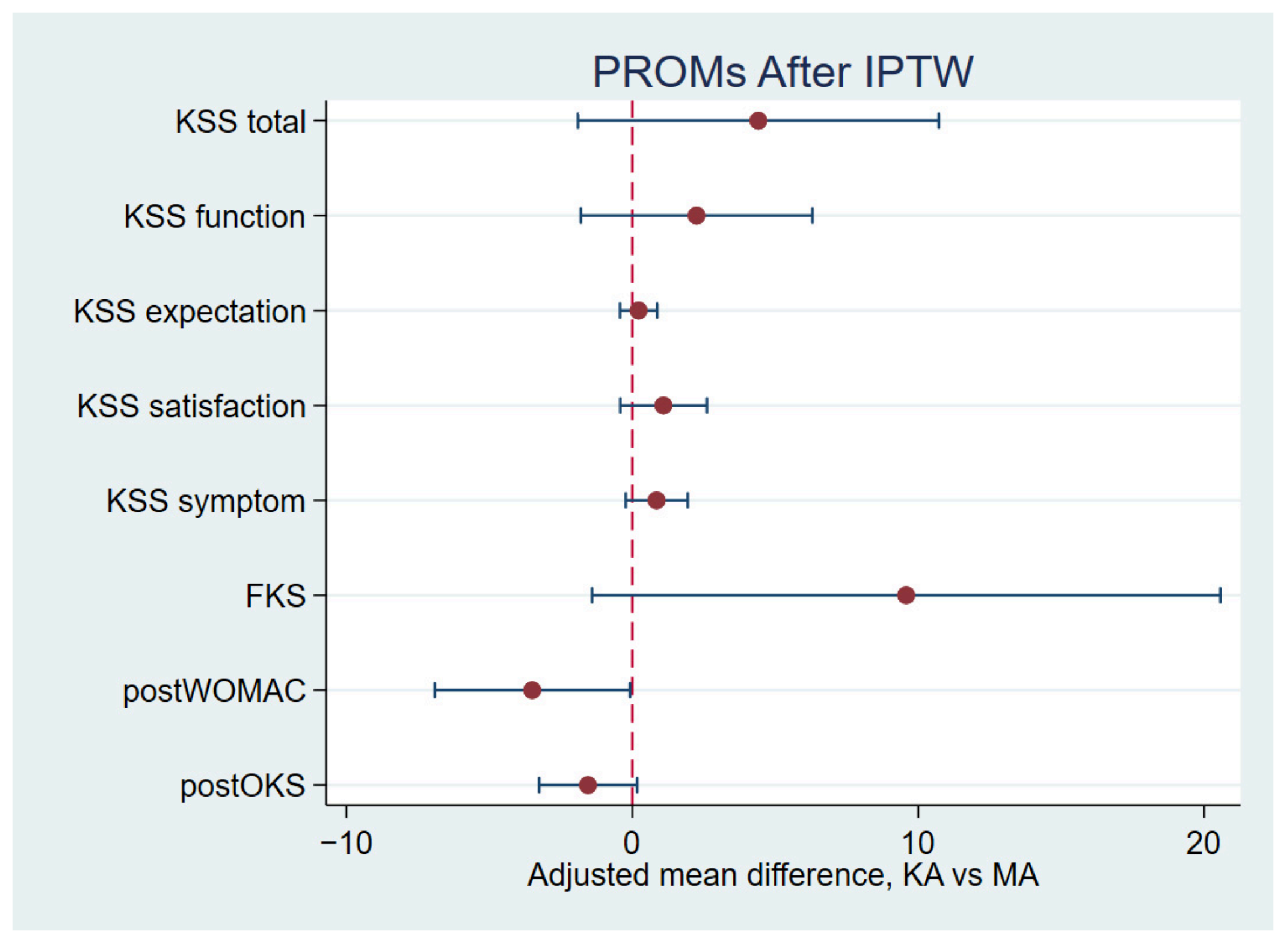
Forest plot of IPTW-adjusted treatment effects for patient-reported outcomes and KSS 2011 domain scores. Points represent adjusted mean differences for KA compared with MA, and horizontal lines represent 95% confidence intervals.

After IPTW with patient-level clustering, none of the patient-reported outcomes reached statistical significance at the two-sided 0.05 level. The adjusted difference in WOMAC was −4.16 points (95% CI, −8.33 to 0.02; *p* = 0.051), while OKS (ATT, −1.89; 95% CI, −3.88 to 0.10; *p* = 0.062) and FJS (ATT, +10.70; 95% CI, −1.31 to 22.71; *p* = 0.081) also did not reach statistical significance. No significant adjusted differences were observed in the KSS symptom, satisfaction, expectation, function, or total patient-reported domains (Table 4) (Figure 10).

### 3.5. Radiographic Outcomes

**Figure 11:**
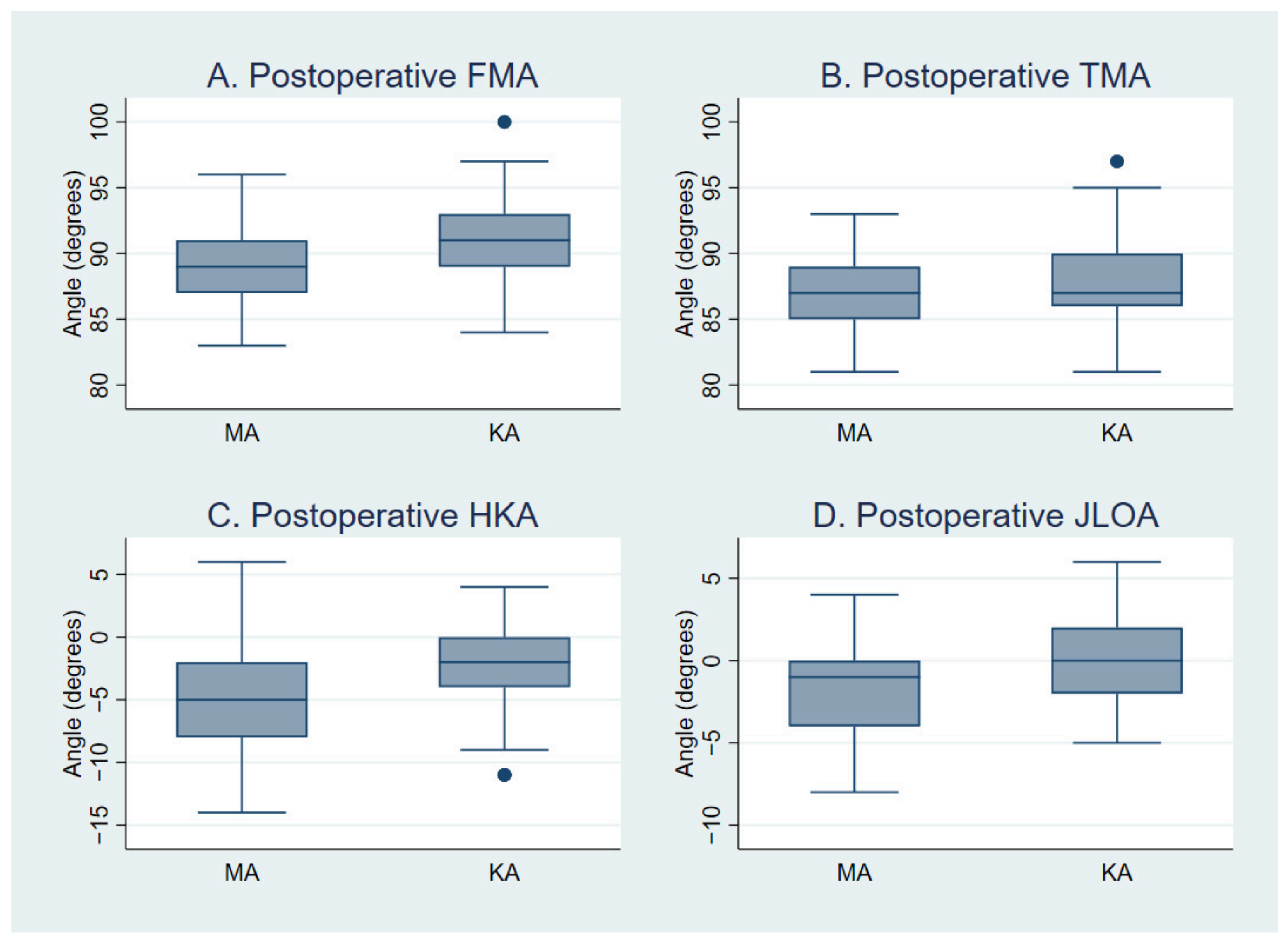
Comparison of postoperative femoral mechanical angle (FMA), tibial mechanical angle (TMA), hip–knee–ankle angle (HKA), and joint line orientation angle (JLOA) between the mechanical alignment (MA) and kinematic alignment (KA) groups. Boxes represent the interquartile ranges (25th–75th percentiles), horizontal lines within boxes indicate the medians, and whiskers extend to the most extreme values within 1.5 times the interquartile range. Dots represent outliers beyond the whiskers.

Postoperative coronal alignment differed between groups. Mean HKA was −2.03 ± 3.15° in KA and −4.61 ± 4.19° in MA. After IPTW adjustment with patient-level clustering, KA was associated with a 2.52° higher HKA (95% CI, 1.15 to 3.89; *p* < 0.001), indicating less residual varus alignment. Major HKA outliers beyond ±6° occurred in 4 of 66 KA knees (6.1%) and 21 of 54 MA knees (38.9%); the adjusted risk difference was −32.92 percentage points (95% CI, −47.33 to −18.50; *p* < 0.001) (Table 5) (Figure 11).

FMA was higher in KA than MA (91.20 ± 3.17° vs. 89.26 ± 3.15°; ATT, +1.92°; 95% CI, 0.80 to 3.05; *p* = 0.001), consistent with a more valgus femoral component orientation. TMA did not differ significantly (87.52 ± 3.09° vs. 86.94 ± 2.76°; ATT, +0.55°; 95% CI, −0.56 to 1.65; *p* = 0.331). JLOA was closer to horizontal in KA (0.21 ± 2.45°) than in MA (−1.74 ± 2.79°), with an adjusted between-group difference of +2.01° (95% CI, 1.05 to 2.98; *p* < 0.001) (Table 5) (Figure 11).

The horizontal line within each box represents the median, the box represents the interquartile range (IQR), and the whiskers represent values within 1.5 times the IQR. Points beyond the whiskers represent outliers.

## 4. Discussion

The principal finding of this study was that modified kinematic alignment (KA) with selective tibial varus restriction was associated with a higher postoperative Objective Knee Society Score 2011 (Objective KSS) and a different coronal radiographic profile compared with historical manual mechanical alignment (MA) controls after inverse probability of treatment weighting. The adjusted difference in total Objective KSS was 15.47 points. The largest component-level difference was observed in alignment (+11.93 points), followed by stability (+2.16 points), whereas the Objective KSS range-of-motion (ROM) component and measured postoperative ROM did not differ significantly between groups. Patient-reported outcomes were largely comparable after patient-level clustering, with no outcome reaching statistical significance at the two-sided 0.05 level. Radiographically, KA was associated with less residual varus HKA, fewer HKA outliers beyond ±6°, a more valgus femoral component orientation, and a more horizontal joint line, whereas tibial component orientation did not differ significantly.

The higher total Objective KSS should not be interpreted as an alignment-independent clinical benefit of KA. Among the surgeon-assessed components, the largest adjusted difference was observed in alignment (ATT, +11.93 points), followed by stability (ATT, +2.16 points), whereas the Objective KSS ROM component did not differ significantly (ATT, +0.37 points; 95% CI, −0.79 to 1.53; *p* = 0.534). Measured postoperative ROM was also similar (ATT, +0.98°; 95% CI, −4.99 to 6.96; *p* = 0.747). These findings indicate that the principal objective signal was related to alignment and, to a lesser extent, stability rather than greater postoperative motion. Importantly, the present data do not justify interpreting the higher total Objective KSS alone as evidence of superior overall patient function.

The surgical strategy evaluated here also requires distinction from both unrestricted KA and restricted-KA protocol. Femoral preparation retained calipered resurfacing principles, but tibial coronal orientation was selectively modified when the medial flexion and/or extension gap remained narrow. The 3D-printed 2° tibial recut guide was required in 37 of 66 KA knees (56.1%): 36 knees underwent one 2° recut, only 1 knee underwent two recuts, and 29 knees did not require the guide.

The radiographic findings are consistent with that technical intent. Postoperative FMA was higher in KA than MA (91.20° ± 3.17° vs. 89.26° ± 3.15°; adjusted difference, +1.92°), whereas postoperative TMA did not differ significantly (87.52° ± 3.09° vs. 86.94° ± 2.76°; adjusted difference, +0.55°; *p* = 0.331). The more valgus-oriented femoral component in the KA group is consistent with the use of calipered femoral resurfacing, which preserves the individualized femoral orientation rather than targeting the mechanical axis. In contrast, tibial orientation was selectively modified only when intraoperative gap assessment indicated persistent medial tightness. Thus, the absence of a significant difference in TMA is compatible with the selective rather than systematic application of tibial varus restriction. JLOA was also closer to horizontal in KA, and major HKA outliers beyond ±6° were substantially less frequent (6.1% vs. 38.9%). The higher rate of residual varus alignment in the historical MA cohort should be interpreted in the context of the practical challenges of achieving neutral alignment with conventional manual MA, particularly in knees with severe varus deformity. Although the theoretical goal of MA is restoration of a neutral mechanical axis, this requires both accurate bone resections and appropriate soft-tissue balancing. In varus knees, extensive medial soft-tissue release may be required to obtain balanced flexion and extension gaps. However, even after release, surgeons may need to adjust the tibial resection, use thicker polyethylene inserts, or accept a degree of lateral laxity to achieve acceptable balance. Because the femoral component orientation in MA is determined by the mechanical axis rather than the patient’s native joint orientation, tibial varus cannot be compensated by the restoration of the constitutional femoral alignment.

Previous studies have demonstrated that the planned neutral alignment target in MA is not always achieved in routine practice. Sappey-Marinier et al. reported that although surgeons aimed for a postoperative HKA between 178° and 182°, more than 40% of knees did not achieve neutral alignment after conventional MA TKA, emphasizing that the intended alignment strategy may differ from the final postoperative result [15]. Therefore, the postoperative HKA distribution observed in our MA cohort (mean HKA −4.61°) may reflect the real-world execution of manual MA in a challenging varus population rather than failure of the MA concept itself.

The difficulty of achieving balanced soft tissues with MA may not be explained solely by inaccuracies in manual instrumentation. In a computer-assisted study by MacDessi et al., in which both KA and MA were performed with navigation, KA achieved a higher proportion of optimally balanced knees than MA across CPAK phenotypes, with the largest differences observed in knees with constitutional varus [16]. In CPAK type I knees, optimal balance was achieved in 100% of KA versus 15% of MA cases, while in type IV knees, the corresponding proportions were 89% and 0%. These findings suggest that the challenge of balancing a varus knee under MA is not simply a consequence of imprecise instrumentation, but may also reflect the mismatch between a neutral mechanical alignment target and the patient’s native coronal alignment and joint-line orientation.

This distinction is relevant to the present study. Our historical MA cohort was performed with conventional manual instruments and had a mean postoperative HKA of −4.61°. Thus, the residual varus observed in our MA group should not be interpreted solely as a technical failure to reproduce the intended neutral target. Rather, in severe varus knees, achieving both neutral mechanical alignment and satisfactory medial–lateral soft-tissue balance may be challenging without substantial soft-tissue or bone-resection adjustments. The present KA technique approaches this problem differently by preserving the individualized femoral orientation according to calipered resurfacing principles while selectively restricting excessive tibial varus. In varus knees, the relatively more valgus-oriented femoral component associated with KA may partially compensate for the varus tibial orientation while preserving the native joint-line obliquity. The present study therefore suggests that a manual KA workflow combining calipered femoral resurfacing with selective tibial varus restriction may provide a practical approach to achieving individualized alignment in settings where navigation or robotic assistance is not routinely available.

The patient-reported outcomes provide a complementary perspective. After accounting for patient-level clustering, none of the PROMs reached statistical significance at the two-sided 0.05 level. WOMAC showed a modest directional difference favoring KA (ATT, −4.16; 95% CI, −8.33 to 0.02; *p* = 0.051), but this did not meet the prespecified threshold for statistical significance. OKS and FJS were likewise not significantly different between groups. These findings should be interpreted in the context of previous studies. Wang et al. reported better medium-term WOMAC and OKS after KA in a meta-analysis of randomized trials, although these differences were not maintained at longer follow-up and FJS was similar between strategies [17]. More recently, Stöve et al. found no significant differences in KSS, OKS, WOMAC, or FJS after restricting their secondary meta-analysis to true unrestricted KA and avoiding duplicate cohort contributions; the certainty of evidence was low or very low [18]. Overall, the present findings are consistent with the inconsistent literature, and a reproducible patient-perceived advantage of KA across outcome domains remains unestablished.

Taken together, the objective and patient-reported findings suggest that the effect of changing the alignment workflow may be more readily detected in alignment-related and examiner-assessed outcomes than in patients’ perception of the replaced knee during the present follow-up interval. This distinction is clinically relevant. A more individualized femoral orientation, a more horizontal reconstructed joint line, fewer major coronal outliers, and a small improvement in measured stability do not necessarily translate into proportionate changes in OKS, FJS, satisfaction, or overall function. PROMs are influenced by multiple determinants beyond implant alignment, including preoperative expectations, pain from other musculoskeletal conditions, rehabilitation, activity level, and psychosocial factors. Therefore, the absence of broad PROM differences should not be viewed as contradictory to the radiographic findings; rather, it indicates that the two outcome domains measure different consequences of TKA.

This study has several limitations. First, the study was not randomized: the MA group was a historical control, whereas the KA group was prospectively enrolled. IPTW can balance only measured covariates and cannot eliminate unmeasured or time-dependent confounding; the reported effects should therefore be interpreted as associations rather than causal treatment effects. Second, although clinical outcomes were assessed by an independent evaluator, blinding to the alignment strategy was not formally documented. Radiographic measurements were performed once by a single experienced radiologist, and inter- or intra-observer reproducibility was not assessed. Third, the historical and prospective cohorts may differ in temporal context despite being treated at the same institution by the same experienced surgeon and using the same implant system and perioperative care protocol. Finally, although follow-up duration was similar between groups, the available follow-up was limited to 12–24 months, precluding assessment of longer-term functional outcomes, implant survivorship, polyethylene wear, aseptic loosening, or revision risk.

## 5. Conclusions

The modified kinematic alignment technique using conventional mechanical instruments and selective tibial varus restriction with a standardized 3D-printed recut guide was feasible and was associated with a higher postoperative Objective KSS and fewer postoperative HKA outliers than historical manual mechanical alignment. The radiographic pattern—higher FMA, similar TMA, and a more horizontal joint line—was compatible with the preservation of individualized femoral resurfacing combined with selective tibial control. However, patient-reported outcomes were largely comparable between groups. The potential practical value of this approach warrants further comparative studies with longer follow-up. These findings should not be interpreted as evidence of superiority over contemporary mechanical alignment techniques, particularly those incorporating computer-assisted navigation or robotic assistance.

## Figures and Tables

**Table 1:** Baseline characteristics before IPTW.

Characteristic	MA (*n* = 54)	KA (*n* = 66)	*p*
Age, years	65.3 ± 8.2	67.2 ± 7.6	0.185
BMI, kg/m^2^	25.7 ± 2.8	25.6 ± 2.6	0.880
Preoperative HKA, °	−13.7 ± 8.9	−13.9 ± 6.5	0.905
Female, *n* (%)	44 (81.5)	51 (77.3)	0.572
Male, *n* (%)	10 (18.5)	15 (22.7)	
ASA I, *n* (%)	5 (9.3)	1 (1.5)	0.111
ASA II, *n* (%)	44 (81.5)	55 (83.3)	
ASA III, *n* (%)	5 (9.3)	10 (15.2)	

**Table 2:** Covariate balance before and after IPTW–ATT.

Baseline Covariate	SMD Before IPTW	SMD After IPTW	Weighted Variance Ratio
Age	+0.244	+0.086	0.894
Male sex	+0.103	+0.099	1.155
BMI	−0.028	−0.034	0.776
Preoperative HKA	−0.022	−0.061	0.593
ASA II	+0.048	−0.018	1.033
ASA III	+0.179	+0.020	1.040

Note. Signed standardized mean differences are reported. All six weighted absolute SMDs were below 0.10. Variance ratios describe residual differences in dispersion; preoperative HKA had a weighted variance ratio of 0.593. MA = mechanical alignment; KA = kinematic alignment; ATT = average treatment effect on the treated.

**Table 3:** Objective KSS and range-of-motion outcomes.

Outcome	MA	KA	IPTW-ATT	95% CI	*p*
Objective KSS 2011	75.76 ± 19.14	90.02 ± 12.74	+15.47	9.07 to 21.87	<0.001
Objective KSS surgeon-assessed	53.44 ± 18.28	67.03 ± 12.71	+14.46	8.34 to 20.58	<0.001
Objective KSS alignment	8.80 ± 17.62	20.23 ± 12.10	+11.93	5.93 to 17.94	<0.001
Objective KSS stability	22.78 ± 4.31	24.62 ± 2.20	+2.16	0.54 to 3.79	0.009
Objective KSS ROM	21.87 ± 3.22	22.18 ± 2.52	+0.37	−0.79 to 1.53	0.534
Postoperative ROM, °	112.04 ± 16.56	112.73 ± 11.13	+0.98	−4.99 to 6.96	0.747

**Table 4:** Patient-reported outcomes after IPTW adjustment.

Outcome	MA	KA	IPTW-ATT	95% CI	*p*
OKS	20.02 ± 5.72	18.89 ± 3.80	−1.89	−3.88 to 0.10	0.062
WOMAC	10.33 ± 12.05	8.18 ± 7.96	−4.16	−8.33 to 0.02	0.051
FJS	73.19 ± 32.72	83.86 ± 26.30	+10.70	−1.31 to 22.71	0.081
KSS symptom	22.31 ± 3.26	22.98 ± 2.46	+1.01	−0.21 to 2.23	0.106
KSS satisfaction	30.48 ± 4.29	31.42 ± 4.28	+1.23	−0.40 to 2.86	0.140
KSS expectation	12.48 ± 1.87	12.65 ± 1.71	+0.31	−0.42 to 1.04	0.402
KSS function	60.37 ± 14.56	61.30 ± 9.58	+2.62	−2.31 to 7.55	0.298
KSS total	125.65 ± 21.65	128.36 ± 15.59	+5.17	−2.36 to 12.70	0.179

**Table 5:** Radiographic outcomes after IPTW adjustment.

Outcome	MA	KA	IPTW-ATT	95% CI	*p*
Postoperative HKA, °	−4.61 ± 4.19	−2.03 ± 3.15	+2.52	1.15 to 3.89	<0.001
HKA outlier > ±6°, *n* (%)	21/54 (38.9)	4/66 (6.1)	−32.92 pp	−47.33 to −18.50	<0.001
Postoperative FMA, °	89.26 ± 3.15	91.20 ± 3.17	+1.92	0.80 to 3.05	0.001
Postoperative TMA, °	86.94 ± 2.76	87.52 ± 3.09	+0.55	−0.56 to 1.65	0.331
Postoperative JLOA, °	−1.74 ± 2.79	0.21 ± 2.45	+2.01	1.05 to 2.98	<0.001

## Data Availability

The data that support the findings of this study are available from the corresponding author upon reasonable request.
